# Effect of 12-week head-down strong abdominal breathing on cognitive function in patients with stable chronic obstructive pulmonary disease: a single-centre randomised controlled trial protocol

**DOI:** 10.1186/s13063-024-08193-8

**Published:** 2024-05-30

**Authors:** Feiyun Song, Kexin Ding, Mingyun Sun, Rui Xia

**Affiliations:** 1 Department of Sports Rehabilitation, School of Physical Education, Anqing Normal University, Anhui, China; 2 School of Physical Education of Chaohu University, Anhui, China

**Keywords:** Abdominal breathing, Cognition, Ba Duan Jin, COPD, Rehabilitation training

## Abstract

**Background:**

Patients with chronic obstructive pulmonary disease (COPD) often suffer from a combination of mild cognitive impairment (MCI) and a significant reduction in their quality of life. In the exercise programme of pulmonary rehabilitation (PR), pulmonary rehabilitation intervention is often carried out by enhancing respiratory function. Strong abdominal breathing is a kind of breathing method, through which the diaphragm can be exercised, thereby enhancing the deflection distance of the diaphragm during breathing and improving respiratory function. The inversion trainer can meet the different angles of head-down training and also has the characteristics of low cost, easy to operate, and use a wide range of scenarios. According to currently available data, strong abdominal breathing in combination with head-down position has not yet been used in pulmonary rehabilitation in this type of rehabilitation programme. It is valuable to use this device to study PR of cognitive function in patients with COPD.

**Methods:**

This study was a 12-week single-centre randomised controlled trial and blinding the assessors and data processors of the test. Recruitment is planned for January 1, 2024. It is expected that 81 patients with stable COPD combined with MCI will be recruited and randomly assigned to the head-down strong abdominal breathing group (HG), the fitness qigong eight-duanjin group (BDJ), and the control group (CG) in a 1:1:1 ratio. Using fNIRS (functional near-infrared spectroscopy) to assess brain oxygen availability before and after pulmonary rehabilitation in three periods: before, during and after the intervention. Cognitive functioning is also assessed using the Overall Cognitive Assessment Scale, the Specific Cognitive Functioning Assessment Scale and the Cognitive Behavioural Ability Test.

**Trial registration:**

The Specialised Committee on Scientific Research and Academic Ethics of the Academic Committee of Anqing Normal University approved the project (ANU2023001). China Clinical Trial Registry approved the study (ChiCTR2300075400) with a registration date of 2023/09/04.

**Discussion:**

The aim of this study was to explore novel exercise rehabilitation methods to improve cognitive function in COPD patients. It results in a lower financial burden and higher participation in pulmonary rehabilitation and improves the quality of survival of patients with COPD.

**Supplementary Information:**

The online version contains supplementary material available at 10.1186/s13063-024-08193-8.

## Introduction

Chronic obstructive pulmonary disease (COPD) is a common preventable and treatable chronic disease characterised by airway obstruction, which is caused by exposure to inhaled particulate matter (e.g. cigarette smoke and air pollutants) as well as genetic, developmental and social factors [1]. In patients with chronic pulmonary obstructive disease, as the disease progresses, stiffness of the soft tissues and joints in the neck and shoulders restricts physical movement, including head extension, high tension in the neck muscles [2], thoracic kyphosis and internal rotation of the shoulders, the contractile properties of the diaphragm fibres are diminished, elasticity is reduced, series of muscle segments are absent, myofibrillar proteins are destroyed and the diaphragm morphology is altered [3]. The diaphragm is an important respiratory muscle for maintaining respiratory ventilation. The mechanical effects caused by systemic inflammation and hyperinflation can lead to diaphragmatic dysfunction in patients with COPD [4]. These changes put the diaphragm at a mechanical disadvantage, resulting in a reduced ability of the diaphragm to generate flow and pressure, increasing respiratory work and leading to diaphragmatic fatigue [5]. Moreover, these changes can cause chest tightness, inspiratory muscle weakness, increased respiratory resistance, severe breathing difficulties and even affect physical activity and the ability to walk [6]. Also, patients with COPD often have comorbid MCI and a higher prevalence of cognitive impairment (range 10–61%, mean 36%) than the normal population (range 5–24%, mean 12%) [7]. It is usually characterised by frequent memory loss, functional disability, poor physical and mental health and rapid decline in cognitive function over time [8]. The aetiology of cognitive impairment in COPD patients is multifactorial, such as lung inflammation (elevated C-reactive protein levels), and hypoxaemia [9, 10].

Although cognitive dysfunction is associated with quality of life and rehabilitation outcomes in COPD patients [11, 12], it has attracted the attention of experts and scholars in the field. However, cognitive rehabilitation therapy for COPD patients is still in the exploratory stage internationally. Currently, VR-based technology combined with health education [13], cognitive therapy based on mindfulness-based therapy [14], pulmonary rehabilitation combined with cognitive training [15], long-term oxygen therapy [16], and lung volume reduction surgery have been applied in the cognitive rehabilitation process of COPD patients [17] and have significant effects. If we look at the ease, economy, and flexibility of implementing cognitive rehabilitation therapy, it seems that the exercise approach through rehabilitation at home or in the community is a good choice. However, the variety of exercise rehabilitation therapies to improve cognitive function in COPD patients needs to be further enriched, so as to provide patients with more diversified choices of exercise rehabilitation programmes.

With the advent of inversion devices in rehabilitation, more and more people are using such devices for rehabilitation. In a study of head-down training in an elderly population, it was shown that changes in body position had an effect on cerebral blood flow and also led to a reduction in psychological stress and improved sleep quality in the lives of older people [18]. In another study on the magnitude of diaphragmatic excursions in healthy subjects, the optimal change in diaphragmatic excursions was obtained in healthy subjects using a 30° head-down tilt position [19]. Strong abdominal breathing is one type of breathing. Currently, studies exploring MCI in COPD patients through head-down strong abdominal breathing training are rare internationally. Previous studies have found that head-down training has a relatively significant effect on, for example, cerebrovascular function [18], while studies of lung function have found heterogeneity. A review of the relevant literature also revealed that respiratory training can improve cognitive function in COPD patients [20], but the depth of research needs to be further improved. Therefore, this study will explore the cerebral blood flow alteration status through head-down strong abdominal breathing training. We also monitored the status of HbO2 changes in the prefrontal cortex by fNIRS and evaluated the improvement of cognitive function in COPD from multiple perspectives by combining the Overall Cognitive Assessment Scale, the Specific Cognitive Assessment Scale, and the Cognitive Behavioural Ability Level Test [21–23].

The effectiveness of traditional Chinese sports in promoting health is well documented. In particular, the Eight Duan Jin exercises have been shown to improve the respiratory function of the body, mainly by enhancing the elasticity of the diaphragm and increasing the volume of the thorax, thereby increasing the inspiratory volume of the lungs and improving exercise capacity [24, 25]. We compared Fitness Qigong Ba Duan Jin with head-down strong abdominal breathing to verify the effectiveness of head-down strong abdominal breathing in relation to traditional Chinese exercise programmes in improving cognitive function in COPD patients.

The design of this study takes into account the significance and value of the practical application of multiple aspects of the study. Firstly, from the patient’s point of view, COPD patients suffer from a range of personal, family and social problems caused by cognitive dysfunction. For example, it reduces the quality of patients’ survival and imposes a huge economic burden on their families and society [26]. Secondly, from the perspective of researchers in related fields, exploring the rehabilitation effect of cognitive function in COPD patients through the exercise rehabilitation of head-down strong abdominal breathing may have a certain reference and borrowing value, so as to facilitate related professionals to explore more scientific and efficient exercise rehabilitation therapies. Finally, from a health system perspective, this study has a relatively large population and scope of application and can be intervened in the home, the community, and in outpatient settings. It may be possible to alleviate some of the pressure on the health care system.

## Research methodology

### Design

**Fig. 1:**
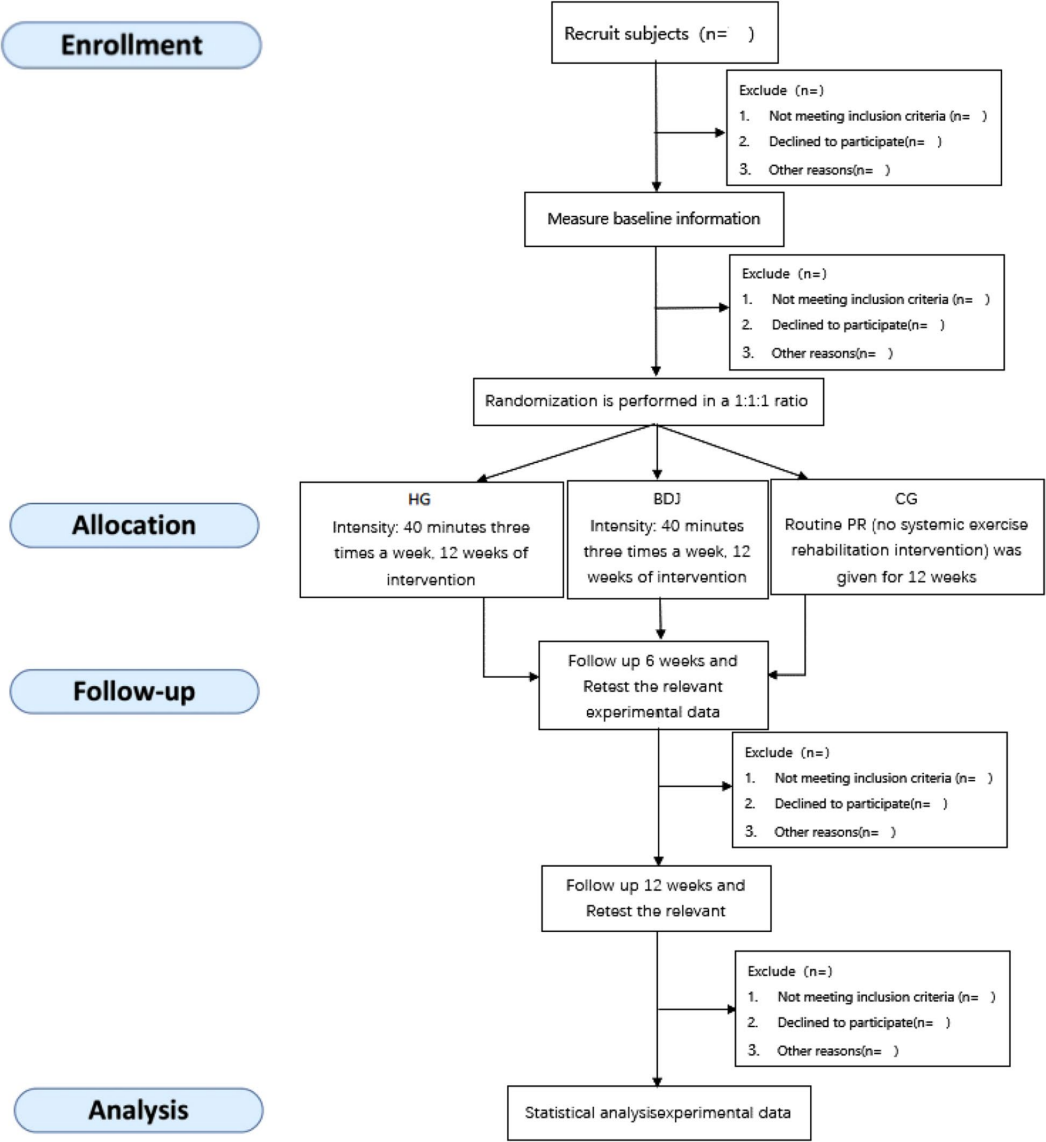
Flowchart of the design of experiments

This study was a single-centre randomised controlled study with three measurement periods (One week before the experiment, week 6 of the experiment and the first week after the experiment.). Assessors for cognitive and physical tests and data analysts are formally blinded, while participants and trainers are unblinded. Eighty-one COPD patients (GOLD I-II) will be recruited for this study and randomly assigned in a 1:1:1 ratio to the HG, BDJ and CG groups, with the HG and BDJ groups receiving a 12-week motor rehabilitation intervention. The availability of oxygen in the dorsolateral prefrontal cortex (PFC) was monitored by fNIRS at all times during the cognitive task, and cognitive function was assessed in conjunction with relevant cognitive scales. To explore the improvement of cognitive function in COPD patients by strong abdominal breathing in a head-down position. This study design and participant flow is shown in Fig. 1, and the relevant demographic and baseline data indicators to be measured are shown in Table 1. For the study duration and SPIRIT checklist, please see the Additional file.

**Table 1:** Demographic and baseline characteristics

Characteristics	HG (*n* =)	BDJ (*n* =)	CG (*n* =)	*P* value
Women, *n* (%)	x	x	x	x
Age, years, ± SD	x	x	x	x
BMI, kg m^2^	x	x	x	x
FEV1, %pred	x	x	x	x
FVC, %pred	x	x	x	x
PaO_2_, mmHg	x	x	x	x
PaCO_2_, mmHg	x	x	x	x
SPO_2_, %	x	x	x	x
GOLD Stage I	x	x	x	x
GOLD Stage II	x	x	x	x
TSI, index	x	x	x	x
SGRQ, score	x	x	x	x
MMRC, index	x	x	x	x
MIP, cmH2O	x	x	x	x
MEP, cmH2O	x	x	x	x
CAT, score	x	x	x	x
6MWD, m	x	x	x	x
Overall cognitive function				
MoCA, score	x	x	x	x
Stroop, index	x	x	x	x
ACE-R, score	x	x	x	x
MMSE, score	x	x	x	x
Specific cognitive functions				
TMT-A, score	x	x	x	x
TMT-B, score	x	x	x	x
Digital breadth test DST, score	x	x	x	x
VFT language fluency test, score	x	x	x	x
Berg Balance Scale (BBS), score	x	x	x	x
Eliminate test, score	x	x	x	x
Cognitive behavioural assessment				
PCI, score	x	x	x	x
TUGT, s	x	x	x	x
30-m pace, m/s	x	x	x	x
30-m walking time, s	x	x	x	x
30-m cadence, steps/min	x	x	x	x

Data are mean ± SD unless specified otherwise. Level of significance was set at *P* ≤ 0.05

### Patient and public involvement

No patients/public were involved in the design, implementation, reporting or dissemination plans for our study.

### Organisation and supervision of the experiment

An Experimental Research Validation Team (ERV) was established prior to the start of the experiment. The main members are the designer of this experimental study, the project leader, the chief physician of the Department of Respiratory Medicine, Anqing Hospital of the People’s Liberation Army Navy, and the data analyst. A seminar is held every Friday, focusing on the optimisation of the experimental protocol. At the same time, an Experimental Steering Committee (ESC) will be established. Primarily responsible for pre-lab training, professional guidance and supervision of patients during labs. Among them, the experimental supervisors mainly included professional master’s degree students in sports training, rehabilitation therapists, and outpatient doctors from the respiratory department of the Naval Hospital Anqing. Experimental supervisors included the project leader and the chief physician of the Respiratory Department of the Naval Anqing Hospital. The Experimental Steering Committee will meet fortnightly. Discusses primarily the status of the implementation of the research protocol within the scope of the experimental phase. Includes problems encountered during experimental guidance, problems identified by experimental supervisors, and patient feedback to experimental staff. And to answer and optimise the corresponding questions, so that this study can be completed scientifically, rationally and smoothly.

### Sample size

The sample size is determined primarily on the basis of the key outcome indicators of the trial. The sample size was calculated by applying ANOVA with G-power (3.1.9.7) software, where the effect size was 0.25, *α* was 0.05 and power was 0.8, resulting in a calculated sample size of 69. Considering that there might be 10–15% sample attrition, we planned to invite 81 COPD patients to participate in the trial.

### Participants

#### Entry criteria

1. Diagnosis of COPD according to the Global Obstructive Lung Disease Initiation (GOLD) criteria (exertional expiratory volume in 1 s (FEV1)/exertional spirometry (FVC) < 70%), while in GOLD stages I–II.
2. MMSE score between 23 and 27 (maximum total score is 30, with 23–27 indicating MCI.

#### Exclusion criteria

1. Inability to follow RMT instructions or complete questionnaires for our study due to cognitive impairment.
2. A COPD exacerbation, chronic heart failure, history of stroke, high-risk cardiopulmonary disease or orthopaedic disease within the previous 4 weeks.
3. Diagnosis of lung cancer or history of thoracoabdominal surgery.
4. A body mass index (BMI) of ≥ 30.
5. Inpatients.
6. Pleural disease or thoracic deformity.
7. Visual or hearing impairment.
8. Receiving medication that may cause cognitive impairment.
9. Completed pulmonary rehabilitation within the last 12 months or currently participating in an exercise programme.
10.  Severe cognitive impairment/dementia (Montreal Cognitive Assessment score < 17) or other diagnosed neuropsychiatric symptoms.

### Recruitment and selection

This study will rely on COPD patients associated with the respiratory department of Anqing Hospital of the People’s Liberation Army Navy and COPD combined with CI patients in the surrounding community of this hospital for sample recruitment to ensure adequate sample size, ease of implementation of the relevant experimental protocol and to reduce sample attrition. Recruitment for This study in this experiment will take place on 1 January 2024, and on 1 February 2024, the 12-week experimental period will officially begin. Relevant medical data and screening will be evaluated by a medical professional. Subjects will also sign an informed consent form indicating their voluntary participation in the trial. The protocol follows the Standard Protocol Items: Recommendations for Interventional Trials (SPIRIT) 2013 [27]. A brief SPIRIT flow diagram is shown in Table 2. A populated SPIRIT checklist is provided in the Additional file.

**Table 2:**
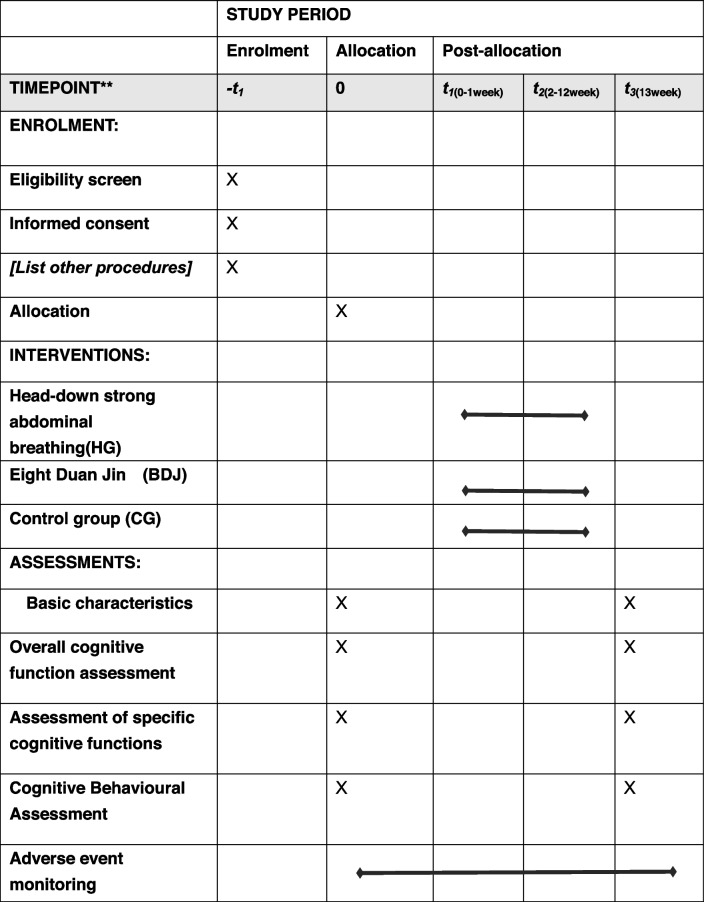
Tabulated summary of the study schedule

### Randomisation and blinding

All participants in the experiment were randomised into groups and sequences will be generated by the random sequence generator (www.random.org) and randomly distributed to the participants by the experimental recruitment team by sealing the sequences in envelopes. The participants were then divided into HG, BDJ and CG groups in a 1:1:1 ratio. Assessors for the cognitive and physical tests were formally blinded, while participants and trainers were not blinded. The data processor will also be blinded to the processing of data reports collected during the pre-, mid- and post-experimental periods and will be required not to communicate with the data processor about the experimental protocol to ensure objective and reliable data analysis. If a patient is unable to accept the group to which he or she has been randomly assigned due to a medical condition, the recruiting team, in collaboration with the physician, will conduct a comprehensive assessment of the patient and document the event. It is also possible to transfer patients to other groups to continue the pulmonary rehabilitation intervention according to their wishes. However, this person’s data on the relevant indicators were not counted in the data analysis pool for this experiment.

### Intervention

#### Head-down strong abdominal breathing group (HG)

**Fig. 2:**
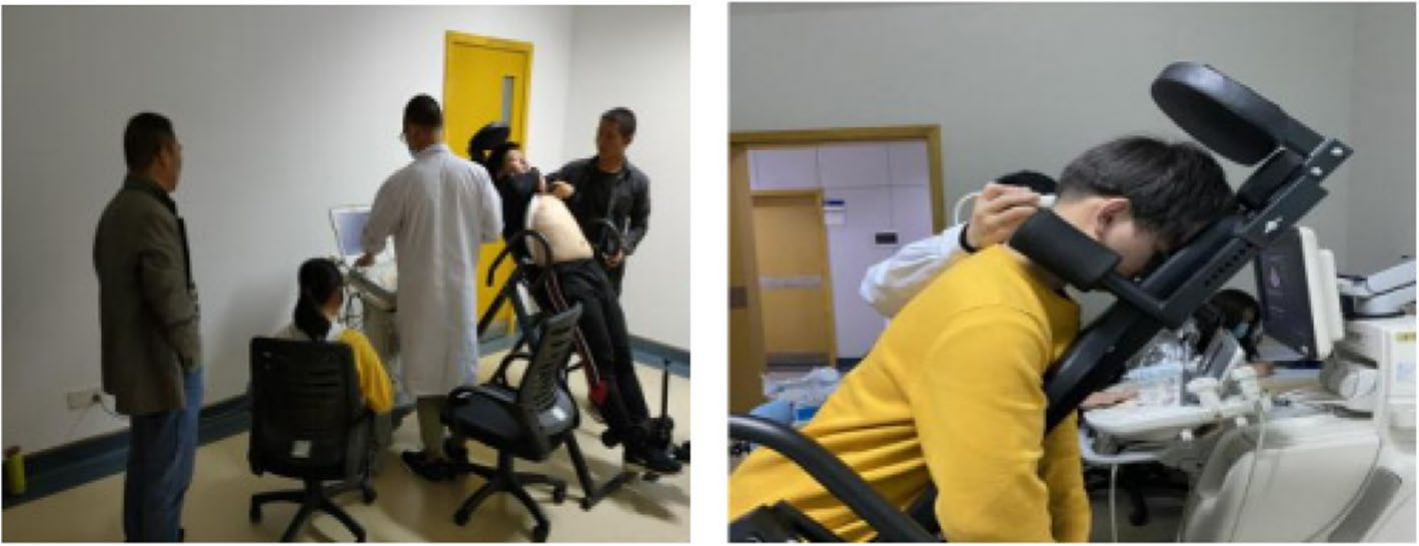
Experimental field diagram of the inverted trainer in different positions

Patients will have a week before the experiment to focus on understanding and learning the head-down strong abdominal exercise to ensure that the participants in the group can perform the relevant movements in a standard way during the formal experiment. During the experiment, head-down strong abdominal breathing will be performed in accordance with the experimental requirements, from easy to difficult, and the participant will be asked to empty their bowels and to perform the head-down strong abdominal breathing at least 1 h after a meal, and to complete the training in one session. The intensity of the intervention was 40 min three times per week, including 5 min of warm-up and acclimatisation at the beginning of the exercise, 30 min of head-down strong abdominal breathing, 5 min of stretching and relaxation, with a respiratory rate of 25 breaths/min (metronome cue), and head-down training on an inverted upside-down dual-purpose exercise machine (Patent No. 201821510570.5) at an inclination angle of − 30°. The intervention period was 12 weeks. Figure 2 shows the field pictures of the carotid blood flow characteristics and the factors influencing it in different positions in young people studied by the head-down strong abdominal breathing in the early stage of this research project [28].This study will also use this inverted upside-down dual-purpose exercise machine to perform head-down strong abdominal breathing exercises.

#### Eight Duan Jin Group (BDJ)

**Fig. 3:**
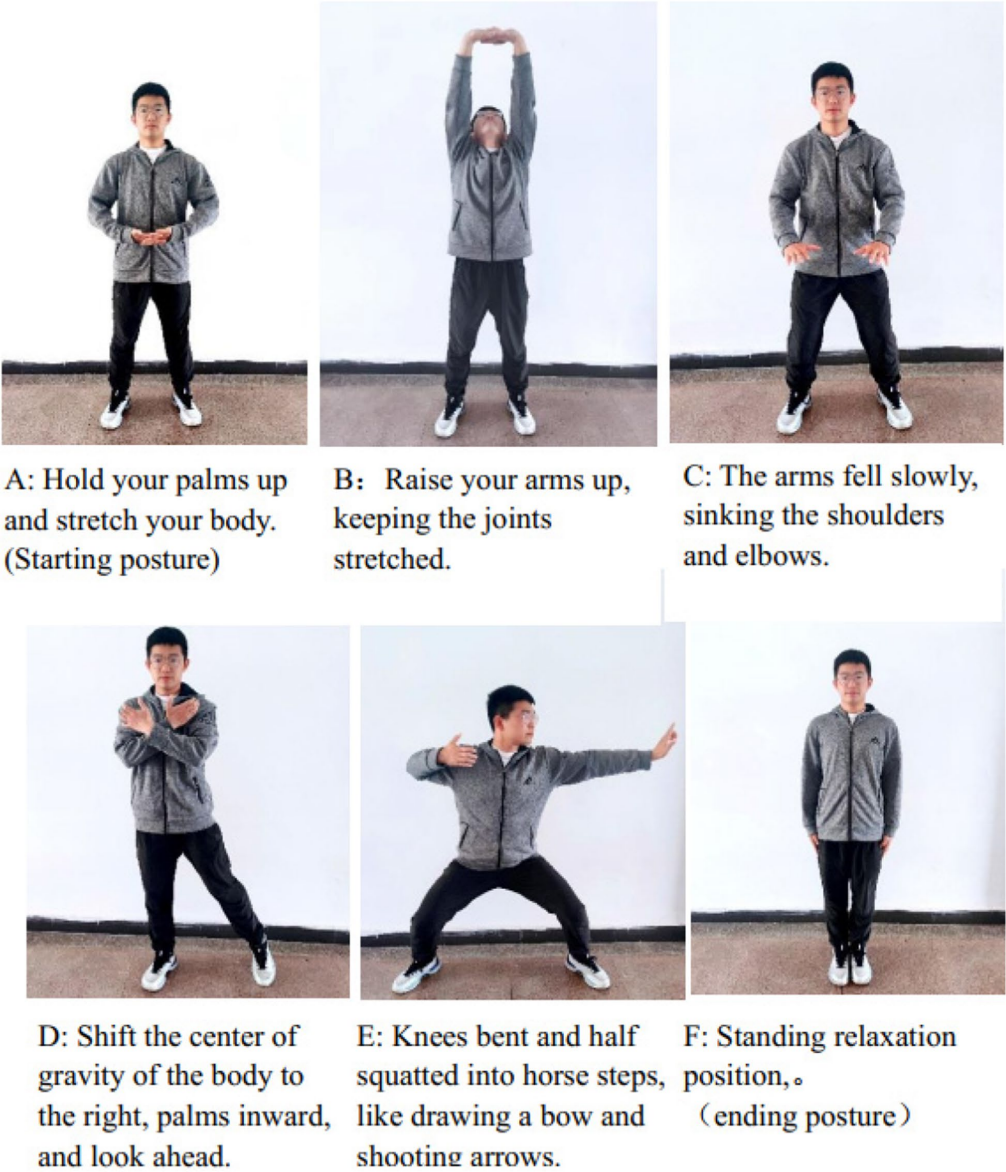
Fitness Qigong eight-dan jin selection movements

The two sets of movements in the Eight Duan Jin of Fitness Qigong, which have a regulatory effect on the respiratory function, were selected: “Two hands resting on the sky to manage the three jiao, and left and right opening the bow like a shooting eagle” [29, 30]. Each session is 40 min and consists of a 5-min warm-up adaptation at the beginning of the exercise, 30 min of head-down strong abdominal breathing exercises and 5 min of stretching exercises to relax, three times a week for a 12-week intervention cycle. The main technical movements are shown in Fig. 3.

#### Control group (CG)

Patients do not undergo any systematic exercise rehabilitation and only undergo a routine medication programme.

### Study results

#### Basic features

##### Lung function

Clinicians will use the hospital respiratory spirometer (Germany JAEGER-MasterScreen) to measure FEV1 (forced expiratory volume in 1 s), FVC (forced vital capacity), FEV1% predicted, FEV1/FVC ratio, MIP (maximum inspiratory pressure), MEP (maximum expiratory pressure), DLco (diffusing capacity of the lung for carbon monoxide), and DLco/VA in COPD patients. The variability characteristics of blood oxygen saturation (SpO2) and other relevant indicators measured by nail oximetry in humans(BERRY® BM1000F). Blood gas indicators such as oxygen saturation (SaO2) and partial pressure of oxygen (PaO2) were measured using a blood gas system (Siemens-RAPIDLab® 348EX) to assess blood gas indicators before and after the intervention, thereby assessing respiratory muscle strength, cardiopulmonary endurance and other functions in COPD patients [31, 32].

##### Respiratory distress symptoms

The subjective severity of the subject’s dyspnoea will be measured using the modified Medical Research Council Dyspnoea Scale (mMRC), the St. George’s Respiratory Questionnaire (SGRQ). The mMRC is a 5-point scale (0–4), with higher scores indicating worse dyspnoea. The SGRQ is a disease-specific questionnaire consisting of three domains (dyspnoea, impact and activity). Each score ranges from 0 to 100 (0 = no impairment), with higher scores indicating severe respiratory symptoms.

### Main results

#### Prefrontal cortex tissue oxygen saturation index (TSI)

Monitoring changes in dorsolateral prefrontal cortex (PFC) oxyhemoglobin concentration by fNIRS at all times during a cognitive task. fNIRS is a powerful tool for the non-invasive study of task-evoked brain activity, allowing real-time monitoring of changes in the concentration of oxyhemoglobin content on the prefrontal cortex during the task [33]. The frontal cortex tissue oxygen saturation index (TSI) is calculated as the ratio of oxy-Hb to the percentage of Total-Hb and reflects the balance between oxygen delivery and utilisation. In the brain, TSI is used as a proxy for tissue oxygenation. Neural activity triggers local changes in cerebral haemodynamics, which induce enhanced blood flow to activated brain regions (neurovascular coupling) [34–36]. It mainly proceeds due to the local oxygen supply being greater than its consumption and therefore higher concentrations of oxyHb and reduced concentrations of deoxyHb are observed in the activated brain regions.

**Fig. 4:**
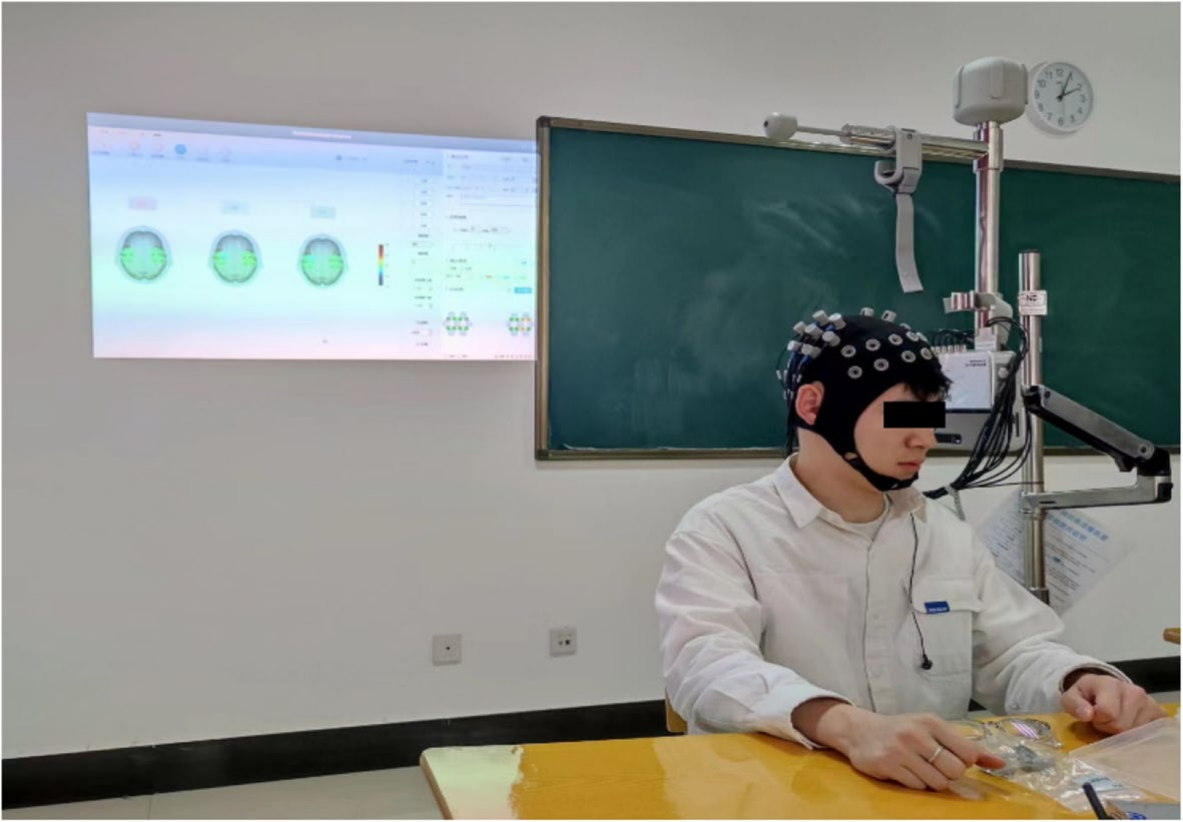
fNIRS test site diagram in task status

For the measurement of the TSI, This study was designed to use a single-task walking test in two contexts. Also record the step frequency, step speed and walking time during the period. The first scenario was a single-task walk at a general pace (6 round trips on a 5-m trail analysis system for a total of 30 m). The general speed of the stride is based on the speed of walking at home. The second scenario is a fast walking single-task walk (6 round trips on a 5-m trail analysis system for a total of 30 m)[37, 38]. A fast walk is a guideline that mimics the speed of a fast walk through a junction. The status of altered cognitive function under the different intervention modalities was assessed by comparing oxygen saturation in the prefrontal cortex before and after the intervention, and by combining indicators such as step frequency, step speed and walking time during the period [39, 40]. In order to minimise experimenter-related methodological bias, the same assessor administered the same instructions to each subject. Each subject completed the task twice, once for familiarisation and once for assessment. Figure 4 shows the fNIRS instrument (portable NirSmart II-3000B Huitron Medical Devices Ltd.) that will be used in this study. It is possible to perform the relevant motor tasks in wireless transmission mode and record the cortical changes in oxyhaemoglobin during the performance of the task in continuous wave form. The light sources are fixed to the head cap at a distance of 30 mm, with a full channel sampling frequency of 11 Hz and wavelengths of 760 nm and 850 nm, respectively, with the detection area being the centre of each light detection point, and with specific brain area cues on the device to ensure accuracy of brain area monitoring.

### Secondary results

It mainly includes assessment results in the cognitive domain (reaction time and accuracy, attention, memory, executive ability, fluency) and tests of cognitive behavioural ability levels.

#### Overall cognitive functioning was assessed using the following methods

- The Montreal Cognitive Assessment Scale (MoCA), a test that includes 11 items on 8 cognitive domains including attention and concentration, executive function, memory, language, visual structure skills, abstract thinking, computation and orientation. With a total score of 30 and a normal score of ≥ 26, its high sensitivity, coverage of important cognitive domains and short test duration make it suitable for clinical use.
- Revised Addenbrooke’s Cognitive Examination (ACE-R (2012)), the ACE-R is a concise cognitive test that assesses the following 5 cognitive domains including attention/orientation, memory, verbal fluency, visuospatial and verbal cognitive abilities. The total score is 100, with the higher the score, the better the cognitive functioning.
- The stroop test measures the patient’s ability to categorise information from the environment and to respond selectively to information. It is used to assess the timing and accuracy of responses before and after pulmonary rehabilitation.
- The Simple Mental State Examination (MMSE) tests for cognitive impairment. The full scale is divided into five cognitive aspects: orientation, memory, attention and calculation, recall and language, and the results are rated on a total score of 30, with higher scores being better.

#### Specific cognitive domains are assessed by the following tests

- The Paddle Elimination Test, a test in which five subtests are completed in 15 min, assesses the subject’s attentional direction and accuracy through the final score and failure rate.
- The Digit Breadth Test DST begins by reading aloud a string of random numbers at a rate of one digit per second and then asking the subject to repeat them out in either sequential or reverse order, with memory levels assessed based on accuracy of completion.
- The TMT Connect Test A-B, which consists of two sub-tests, A and B. The TMT-A tests “visuospatial ability” and “writing motor speed”, while the TMT-B tests “processing speed” and “cognitive flexibility”.
- The VFT verbal fluency test assesses the subject’s verbal ability, semantic memory, etc. The test requires the subject to say as many words of a certain type as possible within a specified time. It can be divided into semantic fluency, phonological fluency and motor fluency, with higher scores being better.
- The Berg Balance Scale (BBS) is examined in 14 items from easy to difficult. Each rated item is divided into five functional levels of 0, 1, 2, 3 and 4 to be scored. The minimum score is 0 and the maximum score is 56. The higher the score the better the balance.

#### Cognitive behavioural ability is measured by the following indicators

- The physical exertion index (PCI) is the difference between the heart rate during the walking task and the heart rate at rest divided by the walking speed. Subjects’ cognitive abilities were assessed by using the PCI index to reflect changes in their cognitive abilities during the motor cognitive task.
- Timed-Stand-Walk (TUGT) is a measure of balance and agility that requires the subject to sit in a chair, time starts, the subject stands, and walks 3 m, then turns 180 degrees to return to the chair and records the time taken.
- The gait test analysis equipment was used to assess the patient’s level of cognitive behavioural ability in terms of changes in step frequency, step speed and walking time before and after the intervention in a 30-m rapid step speed task.

### Safety

During the trial, safety officers will be available to monitor and assist those participating in the trial in real time. Any adverse events will be promptly recorded and reported to the principal investigator who will then decide whether they are related to the trial. When serious adverse events occur, clinicians at the People’s Liberation Army Naval Anqing Hospital will be notified so that they can provide timely treatment. For subjects who withdraw midway through the trial, the investigator must record the reason for withdrawal on the case report form, while maintaining contact with the subject, completing an assessment if possible, and completing an end-of-trial form.

### Experimental environment and data management

The experimental process will rely on community outpatient clinics to provide pulmonary rehabilitation interventions close to the patients, while the monitoring and collection of relevant data will be carried out at the People’s Liberation Army Navy Anqing Hospital. All medical records and assessment data collected from participants will be securely stored on an encrypted mobile hard drive. Only authorised research assistants will have access to the trial data. Blood samples will be stored and processed at the People’s Liberation Army Navy Anqing Hospital laboratory as required.

### Oversight and monitoring

#### Composition of the data monitoring committee, its role and reporting structure

The trial is low risk as the intervention is primarily delivered through exercise rehabilitation therapy accompanied by safety aids. The analyses of the primary outcome indicators will be relatively simple comparisons. An independent Data Monitoring Committee (DMC) was therefore not established.

#### Frequency and plans for auditing trial conduct

Formal trial auditing will not be carried out. This is because it is a low-risk intervention and the establishment of a DMC has not been considered.

#### Plans for communicating important protocol amendments to relevant parties (e.g. trial participants, ethical committees)

Any modification to the protocol, including changes in objectives, design, patient population, sample size and study procedures, required a formal application to the Ethics Committee of Anqing Normal University. If these modifications are approved, they will also be updated in a timely manner on the China Clinical Trials Registry (www.chictr.org.cn). At the same time, we will communicate it to the sponsors, and the investigators.

#### Adherence and adverse events

1. Adherence

Professionals such as exercise rehabilitators, respiratory physicians, nurses, and counsellors will be available to guide patients during the experiment. All patients will be followed up by telephone once a week to ask questions about their feelings, opinions or suggestions about participating in the experiment and to answer them. Also record the subject’s compliance with the trial protocol, including reasons why subjects did not meet the inclusion criteria (e.g. too young, wrong diagnosis); subjects did not complete all of the assigned intervention, or received another intervention, or did not even comply with the experimental intervention at all. The number of subjects who were unable to complete the trial and the reasons for this were recorded during the trial.

(2) Adverse events

The experiment is a low-risk intervention with no serious adverse effects or harm. Any adverse events during the trial, including dizziness, headache, and nausea, will be managed immediately by the healthcare professionals in the on-site research team.

### Provisions for post-trial care

The experiment will be followed by a 6-month period with monthly telephone callbacks. At the same time, the trial was a low-risk intervention with no anticipated harm or compensation for participation.

### Statistical analysis

All statistical analyses were performed using SPSS v.27.0. Data are presented as means and standard deviations or proportions. Standard error of the mean (± SEM) was used to look at trends between cardiorespiratory and cerebrovascular changes during exercise and was more appropriate than SD, which was used to indicate the variability of data between patients. Statistical significance of differences between groups was assessed by analysis of variance, paired *t*-test and chi-square test. In addition, one-way ANOVA with repeated measures was used to determine statistically significant differences between the different percentiles of total exercise duration for oxy-Hb, Deoxy-Hb, Total-Hb, TSI, and blood oxygen saturation during exercise. Multiple comparisons using LSD honestly significant difference post hoc procedure to determine pairwise differences. Pearson’s correlation coefficient was used to assess the bivariate relationship. Multiple linear regression models were used to assess potential relationships between determinants of cognitive impairment and changes in cerebral oxygen utilisation and cerebral haemoglobin volume. Missing values in the data will be statistically analysed by multiple interpolation. The level of bivariate significance was set at *P* ≤ 0.05.

## Discussion

This study will examine the improvement of cognitive function in patients with COPD combined with MCI in terms of physiological indicators and assessment scales. In the past, patients with cognitive impairment have been excluded from PR programmes for COPD patients due to their frequent memory loss, dysfunction, and poor physical and mental health, which hinder the effectiveness of PR interventions [8]. There is a need to explore treatments that can improve cognitive function in COPD patients in order to improve the quality of their survival [41]. Aras et al. [42] showed that the coexistence of sleep disturbance, depression and anxiety with COPD increased cognitive impairment and the severity of the disease. In a study on the relationship between cognitive function and the brain, Savage et al. [43] found in their study that frontal lobe atrophy was significantly greater in COPD patients than in controls (*p* = 0.02) using a visual rating scale, and that cognitive function was significantly worse in the COPD group in terms of executive function (*p* < 0.001), working memory (*p* = 0.02), verbal memory (*p* = 0.03) and processing speed (*p* = 0.001) were significantly worse. In a PR programme for a population of patients with COPD combined with cognitive impairment, Andrianopoulos et al. [44] compared cerebral oxygen availability during exercise in cognitively impaired (CI) and cognitively normal (CN) COPD patients. During exercise, CN and CI patients exhibited mild to moderate decreases in SpO2 (nadir [Δ] ≥  − 3 ± 2% and – 5 ± 3%, respectively) but maintained baseline frontal cortical TSI levels, while presenting small TcPCO2 perturbations and increased total brain-Hb (post [Δ] ≥ 2.0 ± 3 μM s − 1). Patients with CI retain the ability to adequately maintain cerebral oxygen availability during submaximal exercise. Therefore, rehabilitative exercise training for patients with COPD combined with CI who exhibit mild to moderate exercise-induced decreases in SpO2 does not appear to result in reduced cerebral oxygen availability. As in healthy older adults, cardiorespiratory fitness is positively associated with cognitive function in COPD patients [45], and cognitive function in COPD patients can be improved by targeted cardiorespiratory interventions in PR programmes.

Ba Duan Jin is a traditional Chinese sport with a long history, easy to learn and highly effective. The practice of Ba Duan Jin requires no equipment, is not limited by space, and has significant effects on strengthening the body, regulating the movement of qi and blood in the internal organs, and improving respiratory function [46]. Yang et al. [47] concluded from a study of patients with mild COPD that traditional Ba Duan Jin exercises can prevent the deterioration of lung function in COPD and provide a simple, inexpensive, and routine pulmonary rehabilitation measure for patients with mild COPD. This experiment compares the Baduanjin group with the HG group, thus arguing for the effectiveness of the HG training method to be significant.

Strong abdominal breathing, as a form of breathing, intervenes on the respiratory muscles as well as on the carotid blood flow by means of postural adjustments and the diaphragm. In previous studies, head-down training has been found to have a rehabilitative effect in the musculoskeletal system [48], and Su Quansheng et al. [49] showed increased myocardial excitability and improved myocardial contractile performance in the prone and inverted positions in humans. Guo et al. [18] showed that changes in carotid blood flow were related to body position and also had a further contribution to the reduction of psychological stress and improved sleep quality in the lives of older people. However, Guo et al. [50] also studied the head-down bed rest manoeuvre and found that human lung function was not affected. It has also been shown that regular and quantitative inversion training improved the central nervous function of archers and significantly alleviated adverse emotions such as anxiety, poor sleep quality and other discomfort symptoms in good archers after load training [51, 52]. Regarding the control of the angle of the head-down position and respiratory rate, we referred to the angle (0–90°) and respiratory rate (35 breaths/min) of the head-down position performed in the previous period in healthy university students as well as in the elderly. It also combined the characteristics of COPD patients in terms of cardiorespiratory function, etc., the angle of head-down position was proposed to be adjusted to − 30°, and the respiratory rate was proposed to be adjusted to 25 breaths/min. The head-down angle and respiratory rate will also be assessed before the formal experiment, and the angle will be adjusted according to the patient’s actual condition.

For the improvement of cognitive function in patients with COPD combined with MCI, We analysed data on the status of changes in oxyhaemoglobin in the prefrontal cortex of the brain during the task (walking) as detected by fNIRS. The fNIRS analysis only considered the single-task condition, as we expected confounding behavioural differences to occur in the dual-task condition, and therefore the fNIRS analysis was not valid in this condition [53]. However, fNIRS will inevitably generate noise and artefacts during the motion task [54–56], such as breathing, heartbeat, environmental noise and instrument noise, which will interfere with the reliability of the experimental data analysis [57], We will also correct the relevant data. However, in order to enhance the true reliability of the experimental results, we will also assess cognitive functioning more comprehensively with the internationally used Montreal Cognitive Assessment Scale (MoCA), the Revised Addenbrooke’s Cognitive Examination (2012) ACE-R and specific cognitive domains (i.e. attention (Attention Matrix Test), memory (Digital Breadth Test DST), executive ability (TMT Connectedness Test A-B), fluency (VFT)) and cognitive behavioural ability level tests.

In conclusion, this study will explore a new community-based PR programme for patients with COPD combined with IC in order to promote its application in the general population. The HG will be used to intervene in the cognitive function, mental status and motor ability of patients with COPD combined with MCI, and to select a proven intervention that will enrich the current PR programme for COPD.

### Strengths of this study

1. According to currently available information, the HG breathing training method is not found in the PR programme for COPD, and the inverted and upside-down exercise equipment used in this study is a nationally recognised and patented product, whose design and use have been recognised by many. The research project leader is one of the patent holders of the product and has also participated in HG-related research, which will provide more professional guidance and support for this study.
2. This study combines conventional cognitive measurement scales and fNIRS to make the experimental findings more objective and scientific by combining quantitative physiological and biochemical indicators with cognitive assessment.
3. We will explore PR programmes for COPD in the community, where HG training apparatus and equipment are relatively inexpensive and easy to implement for home and community-based organisations.

### Shortcomings of this study

1. Patients need to actively cooperate with each breathing exercise during the performance of head-down strong abdominal breathing, but the implementation process does not allow for precise detection of the patient's breathing pattern, which requires us to enhance the ideological education of the subject prior to the intervention.
2. The assessment results may be influenced by the subjective bias of this researcher, as the blinded intervention was not performed during the administration to the subjects.

## Trial status

Participants will take place from 1 January 2024 to 31 January 2024. This study is expected to be completed by April 2024. This study protocol has been submitted prior to the end of recruitment and prior to the last patient. This study has been registered with the Chinese Clinical Trial Registry on 4 September 2023 (https://www.chictr.org.cn/).

### Supplementary Information

Supplementary Material 1.

## Data Availability

The data analysed during this study are available from the corresponding author upon reasonable request after publication of the article.

## Abbreviations

COPD: Chronic obstructive pulmonary disease
PR: Pulmonary rehabilitation
MCI: Mild cognitive impairment
6MWD: 6-Minute walk distance
MID: Minimal important difference
V̇O2: Peak oxygen uptake
CAT: COPD Assessment Test
SGRQ: St. George’s Respiratory Questionnaire
FEV1: Forced expiratory volume in 1st second
FVC: Forced vital capacity
PaCO2: Partial Pressure of Carbon Dioxide
TSI: Tissue oxygen saturation index in the frontal cortex
MIP: Maximum inspiratory pressure
MEP: Maximum expiratory pressure
MoCA: The Montreal Cognitive Assessment
ACE-III: Addenbrooke’s cognitive examination-III
MMSE: Minimum Mental State Examination
TMT: Trail making test
VFT: Verbal fluency test
HG: Head down strong abdominal breathing group
BDJ: Baduanjin group
CG: Control group
TUGT: Timed up and go test
SpO2: Oxygen saturation
PFC: Prefrontal cortical
